# The academic Great Gatsby Curve

**DOI:** 10.1098/rsif.2024.0173

**Published:** 2024-08-14

**Authors:** Ye Sun, Fabio Caccioli, Xiancheng Li, Giacomo Livan

**Affiliations:** ^1^ School of Mathematics, Southeast University, Nanjing 210096, People’s Republic of China; ^2^ Department of Computer Science, University College London, 66–72 Gower Street, London WC1E 6EA, UK; ^3^ London School of Economics and Political Science, Systemic Risk Centre, London WC2A 2AE, UK; ^4^ London Mathematical Laboratory, London W6 8RH, UK; ^5^ School of Business and Management, Queen Mary University of London, Mile End Road, London E1 4NS, UK; ^6^ Dipartimento di Fisica, Universita degli Studi di Pavia, via Bassi 6, Pavia 27100, Italy

**Keywords:** science of science, mobility, impact inequality

## Abstract

The Great Gatsby Curve measures the relationship between income inequality and intergenerational income persistence. By using genealogical data of over 245 000 mentor–mentee pairs and their academic publications from 22 different disciplines, this study demonstrates that an academic Great Gatsby Curve exists as well, in the form of a positive correlation between academic impact inequality and the persistence of impact across academic generations. We also provide a detailed breakdown of academic persistence, showing that the correlation between the impact of mentors and that of their mentees has increased over time, indicating an overall decrease in academic intergenerational mobility. We analyse such persistence across a variety of dimensions, including mentorship types, gender and institutional prestige.

## 1. Introduction

Intergenerational income mobility, which measures the extent to which income is passed on from one generation to the next, reflects the degree of openness in a society. Lower levels of mobility imply higher class persistence, meaning that an individual’s economic status is largely determined by their family background [1,2]. In recent years, the issues of declining mobility [3] and rising inequality [4], as well as their interrelationship [5], have attracted considerable attention from scholars and policymakers. The empirically observed positive correlation between income inequality and intergenerational income persistence [6,7] is often referred to as the Great Gatsby Curve in the literature, based on the 1925 novel by F. Scott Fitzgerald exploring—among others—the theme of class persistence. Such a relationship has important implications for understanding the mechanisms of social mobility in different contexts, and the potential policy levers to enhance it.

A number of recent studies have investigated concepts that represent the academic equivalents of inequality and intergenerational persistence. The former refers to the uneven distribution of opportunity [8] and academic impact [9,10], which—in spite of its multi-faceted nature—is usually operationalized in terms of the volume of citations accrued by publications over time [11]. The latter is instead quantified by the influence that a mentor’s status may have on their protégés’ academic success [12–15].

In this article, we seek to determine whether an ‘academic Great Gatsby Curve’ exists, i.e. whether academic inequality and intergenerational persistence are positively correlated. In line with the above studies, we quantify academic inequality as the concentration of impact in a population of authors, as measured by the Gini coefficient of the distribution of citations. We operationalize academic intergenerational persistence as the correlation between the academic impact of mentors and that of their protégés, mirroring the association between parents’ and children’s economic well-being. In fact, a mentor can sometimes be seen as a mentee’s ‘academic parent’, as reflected by the German terms for supervisor, Doktorvater or Doktormutter, literally meaning doctoral father or mother. However, unlike the transmission of economic welfare from parents to children, the inheritance between mentors and mentees mainly involves the transfer of research skills and experience, knowledge of the field [16] and professional networks [13–15]. We expect a high level of persistence across academic generations to be associated with unequal opportunities in academia, which we seek to detect as inequality in the distribution of citations across authors in a discipline.

We already documented a positive relationship between academic impact inequality and lack of mobility in impact rankings in a previous study [10]. In that case, the notion of mobility we considered was related to different moments in an author’s career. Here, instead, we are interested in mobility across academic generations and therefore in comparing an author’s academic status with that of their mentors. This is the closest academic equivalent to intergenerational mobility as considered in the social sciences.

In the following, we analyse genealogical data on more than 300 000 academics who published nearly 10 million papers in 22 disciplines from 2000 to 2013 (see §4), examining temporal trends of academic persistence between mentors and their mentees, and comparing such trends across different mentorship types, different mentor–mentee gender combinations and different tiers of institutional prestige. Finally, we document the existence of an academic Great Gatsby Curve, namely the positive relationship between academic impact inequality and academic intergenerational persistence.

## 2. Results

**Figure 1.**
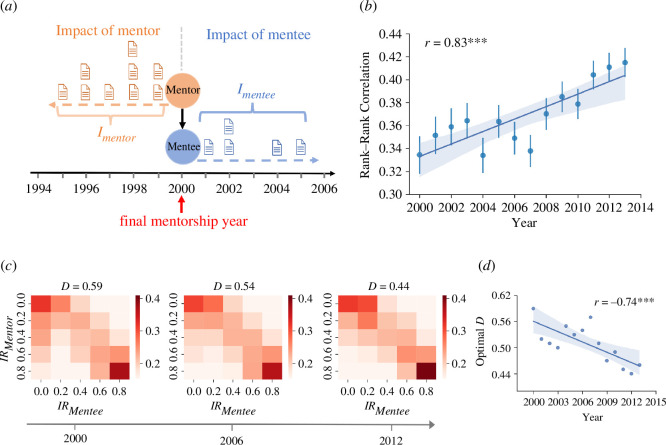
Persistence of academic impact between mentors and mentees. (*a*) Simple illustration of our assumptions to measure the scientific impact of mentors and mentees within a 5-year time window before and after the final mentorship year. The impact of a mentor/mentee over a period of time is calculated as the total number of citations received by their papers (within 5 years after publication) published over that period. (*b*) Persistence of academic impact between mentors and their mentees is on the rise. Here, impact persistence is measured as Spearman’s rank correlation coefficient between the positions of mentors and mentees in the impact rankings of their discipline, using data on cohorts of mentor–mentee pairs with the same final mentorship year. The error bars represent the 95% confidence intervals obtained via bootstrap resampling 5000 times with replacement. (*c*) Evolution of the impact ranking transition matrix between mentors and their mentees. Results are shown for three different cohorts of mentor–mentee pairs with final mentorship years in 2000, 2006 and 2012, respectively. (*d*) The persistence of academic impact across academic generations is increasing over time. This is captured by the decrease in the optimal D parameter estimated from the random walk model. The solid line and the shaded area in (*b*) and (*d*) represent the regression line (with annotated Pearson’s r and p -values) and the 95% confidence interval, respectively. ***p < 0.01, **p < 0.05, *p < 0.1.

Figure 1*a* illustrates how we quantify the academic impact of mentors and mentees within a 5-year time window before and after the final year of their mentor–mentee relationship (hereafter referred to as ‘final mentorship year’), such as, e.g. the year of the mentee’s doctoral graduation. The aggregated impact of a mentor or mentee over this 5-year period is calculated as the sum of the citations received by their papers (within 5 years of their publication) published during such period. To analyse the persistence of impact across academic generations, we calculate the Spearman rank correlation coefficients between the impact percentile ranks of mentors and mentees for cohorts with different final mentorship years. In other words, we measure the similarity between the positions of mentors and their mentees in the impact rankings of their discipline: The higher the rank–rank correlation, the more a mentee’s scientific impact is correlated to that of their mentor, the higher the intergenerational persistence. Figure 1*b* shows a significant upward trend in rank–rank correlations, indicating an increasing trajectory of persistence across subsequent mentor–mentee cohorts. This suggests that over time mentees have become increasingly likely to share similar positions in their discipline’s impact ranking as their own mentors. This finding is consistent with the observation that the academic impact of early-career researchers is increasingly influenced by the prominence and reputation of supervisors [13] and/or collaborators [14], as well as with the existence of a ‘chaperone effect’ in scientific publishing [15]. To verify that this trend is not just an artefact due to our definition of academic impact, we re-evaluate impact after normalizing citations over time and disciplines and re-evaluate the impact of mentors over longer periods of time, i.e. from the year of their initial publication to the final mentorship year, reaching the same conclusion (electronic supplementary material, figure S1). To further test the robustness of our results, we also measure the Pearson correlation between the logarithmic impact of mentors and mentees, once again reaching the same conclusions (electronic supplementary material, figure S2).

To further provide an intuitive description of academic intergenerational persistence across different impact levels, we rank the mentors (mentees) in each cohort based on their aggregated impact over a 5-year period before (after) the final mentorship year and divide both rankings into quintiles. We then construct 5×5 row-stochastic transition matrices, with entries indicating the empirically estimated probabilities that a mentee whose mentor was in a certain quintile of the mentors’ ranking ends up in a certain quintile of the mentees’ ranking. Figure 1*c* illustrates three heat maps of transition matrices for cohorts of mentor–mentee pairs with final mentorship year in 2000, 2006 and 2012, respectively. As one can see, most mentees typically remain in the same quintile as their mentors or move to an adjacent one, a pattern that has manifested with increasing prominence over successive periods. This phenomenon is particularly pronounced among the top- and bottom-ranked mentors, whose mentees show a heightened propensity to remain in a similar ranking position as their mentors. To assess and compare the overall persistence of impact rankings between mentors and their mentees over time, we calibrate a random walk model [10] to the annual transition matrices. Through this procedure, we estimate an optimal value for the model’s diffusion coefficient D , which serves to quantify the extent of impact persistence between mentors and mentees (see §4). A lower D value is associated with higher persistence. Let us note that standard estimators (e.g. the Shorrocks Index [17]) usually quantify mobility in a ranking only through the diagonal entries of a mobility matrix, whereas the D coefficient estimates mobility by considering off-diagonal entries as well. Figure 1*d* shows that overall persistence has steadily increased for cohorts of mentor–mentee pairs with mentorship final years ranging from 2000 to 2013.

**Figure 2.**
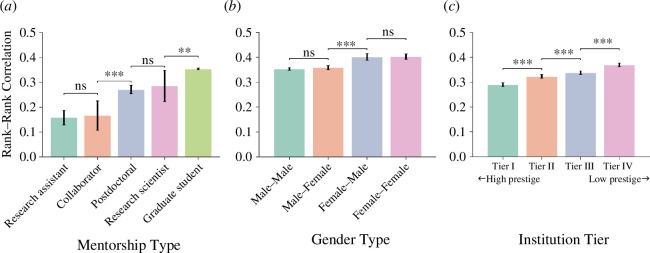
Comparison of impact persistence across different (*a*) mentorship types, (*b*) mentor–mentee gender combinations and (*c*) tiers of institutional prestige. Here, research institutions are stratified into four equal-sized tiers based on the total number of citations received by all papers published by such institutions after the year 2000. The error bars represent the 95% confidence intervals obtained via bootstrap resampling 5000 times with replacement. The results obtained via bootstrap testing for the null hypothesis of equal means between adjacent bars in the histograms are reported on top of the histograms. ***p < 0.01, **p < 0.05, *p < 0.1.

We now proceed to investigate the disparities in impact persistence across various dimensions. We compare persistence across five different mentorship types (figure 2*a*; electronic supplementary material, table S1), finding that research assistants and collaborators display the lowest rank–rank correlations with their mentors, followed by research scientists and postdoctoral fellows. The highest rank–rank correlation (i.e. the highest impact persistence) is observed for graduate students, in line with the expectation that supervisors may have a closer and more supportive relationship with their students, and therefore a stronger influence on their career prospects [13,18]. In addition, we investigate impact persistence between different mentor–mentee gender combinations in figure 2*b*. Our results show a slightly higher persistence associated with female mentors. This is possibly owing to female mentors having a lasting positive impact on mentees [19] or providing career development facilitation to a larger extent than male mentors [20]. After controlling for the mentor’s gender, we find no statistically significant difference in persistence among mentees of different genders. Furthermore, to understand whether intergenerational impact persistence varies according to the prestige of institutions in which the mentorship took place, we first rank institutions based on the total number of citations received by papers published by authors affiliated with them (as a proxy of their prestige) and then divide institutions equally into four tiers based on the quartiles of such ranking. Figure 2*c* reveals that the impact persistence between mentors and mentees is negatively correlated with the prestige of their institution, implying that persistence is relatively lower for top-tier institutions.

**Figure 3.**
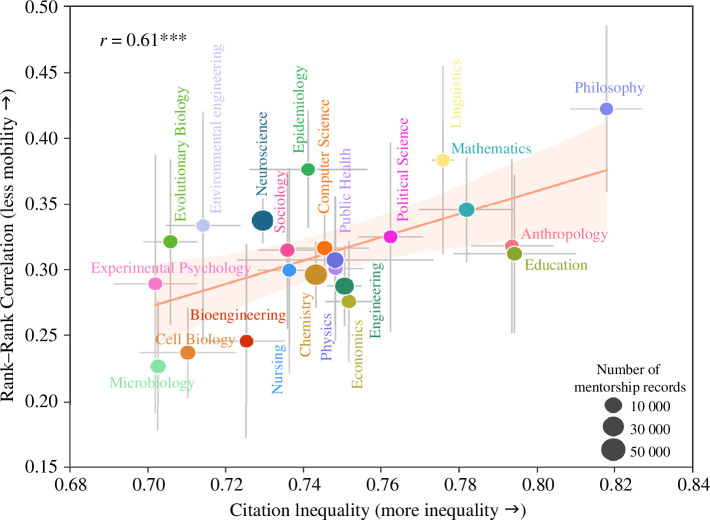
The academic Great Gatsby Curve: More inequality is associated with more impact persistence across academic generations. Citation inequality is measured by the Gini coefficient, using the cumulative number of citations authors (including both mentors and mentees) have received from the papers published within a 5-year time window before the final mentorship year, within 5 years after publication. Mentor–mentee pairs are pooled over time per cohort (with the same final mentorship year) to compute the impact persistence and inequality metrics. The error bars in vertical and horizontal directions refer to the standard deviation of the mean over cohorts. The point size of each discipline is proportional to the number of mentor–mentee pairs considered in our analysis. The solid line and the shaded area represent the regression line (with annotated Pearson’s r and p -values) and the 95% confidence intervals, respectively. ***p < 0.01, **p < 0.05, *p < 0.1.

Inspired by the Great Gatsby Curve in the social sciences [5], we examine the association between impact inequality and intergenerational impact persistence. Figure 3 ranks the disciplines included in our analysis along these two dimensions. The horizontal axis shows the impact inequality in a research discipline, measured by the Gini coefficient of the distribution of citations received by authors from their papers published within a 5-year time window before each final year of mentorship, including both mentors and mentees. Over the past decade, researchers in disciplines such as experimental psychology, microbiology and evolutionary biology experienced the most egalitarian citation distribution, while those in philosophy, education and anthropology the most unequal. The vertical axis shows intergenerational impact persistence, obtained via the Spearman rank correlation as explained above. In disciplines like microbiology, cell biology and bioengineering, the correlation between the impact of mentors and mentees is the weakest. On the contrary, in some liberal arts and medical disciplines like philosophy, linguistics and epidemiology, the persistence of impact across academic generations is rather strong. More importantly, we observe a significant positive correlation between these two quantities (with Pearson’s r=0.61 , p<0.01 ), suggesting that disciplines with greater inequality in their distributions of impact also tend to be areas in which academic impact is more likely to be passed on from mentors to mentees. We ran robustness checks using Pearson correlation instead of Spearman to quantify persistence, finding a consistent positive correlation between impact persistence and inequality (electronic supplementary material, figure S3), with Pearson’s r=0.75 , p<0.01 , and using the D parameter estimated from the random walk model to estimate persistence (electronic supplementary material, figure S4), which we find to be negatively correlated with inequality (Pearson’s r=−0.64 , p<0.01 ) in agreement with other results (as a higher D indicates lower persistence). Furthermore, we have measured the inequality of citation distribution among mentors only and arrived at the same conclusions (electronic supplementary material, figure S5). The values of inequality and the three different measures of academic persistence in each discipline are summarized in electronic supplementary material, table S2. The positive correlation between impact persistence and inequality remains even when controlling for different mentor–mentee gender combinations (electronic supplementary material, figure S6) and levels of institutional prestige (electronic supplementary material, figure S7). Taken together, these results suggest a robust negative association between impact inequality in a given discipline and the potential for its early-career researchers (i.e. mentees) to achieve upward mobility.

## 3. Discussion

In this article, we find that academia is not immune from the phenomenon of intergenerational persistence, which has been widely documented in the social sciences across dimensions such as income, wealth and occupation [1,2]. We examined intergenerational academic persistence by analogizing academic mentors and mentees to parents and children, and academic impact (as measured with citations) to income. The persistence of income through genealogical generations and the persistence of impact through academic ones both reflect the transmission of resources and status, and they capture the extent to which the success of one generation may depend on that of the previous one. However, while there is a clear analogy between the mechanisms at play in these two contexts, there are also obvious differences. On the one hand, both mechanisms involve the inheritance of a network, be it social, professional or both [21,22]. On the other hand, the transfer of economic status is—to a good extent—mechanistic, as it is grounded upon the inheritance of wealth. The transfer of academic status is instead grounded upon the inheritance of intangibles [16], such as knowledge and visibility. Additionally, an important distinction lies in the fact that mentor–mentee pairings are determined by choices made by both parties (as opposed to parents and children). More successful mentors may have the privilege of being more selective in their choice of mentees, and vice versa, leading to a positive correlation between their impact, even in the absence of causal effects.

Let us also acknowledge that our analysis does not and cannot assess how a mentor’s academic status may influence the success of their mentees who leave academia (and who represent a certain portion of the authors in our dataset, electronic supplementary material, figure S8). In this respect, it is only fair to assume that mentees with impactful mentors are likely to have rather successful post-academic careers.

As a limitation to our analysis, we ought to acknowledge that we do not separate—due to lack of information in our dataset—the effect of the prestige of a mentee’s post-mentorship institution from the effect of the mentor’s institution’s prestige. Nevertheless, we have reason to believe that incorporating those effects separately would not qualitatively alter our main findings, as previous research has shown that academic recruitment practices tend to perpetuate pre-existing inequalities [23] and that the transfer of impact from mentors to mentees is significant even after controlling for institutional prestige [14].

Our findings suggest that academia has become less open and more stratified over time, as newer protégé cohorts are characterized by lower intergenerational mobility than their predecessors. We also demonstrated that there are significant differences in impact persistence across different types of mentorship, mentor–mentee gender combinations and levels of institutional prestige.

Finally, we demonstrated the existence of an ‘academic Great Gatsby Curve’, i.e. of a positive relationship between academic impact inequality and intergenerational persistence, in analogy with the Great Gatsby Curve observed between income inequality and intergenerational persistence in the social sciences. This result makes it clear that academic impact—as quantified by citations—is to some extent inherited. As such, citation-based bibliometric indicators should be handled with care when used to assess the performance of academics.

## 4. Material and methods

### 4.1. Dataset

We collected genealogical data on mentorship relationships from the Academic Family Tree (AFT, Academictree.org), including 245 506 mentor–mentee relationships among 304 395 authors who published 9 809 145 papers across 22 disciplines. For each author, we record the person’s ID, name, gender, affiliation and discipline. For each mentor–mentee relationship, we record the IDs of the mentor and mentee, the mentorship type (i.e. graduate student, postdoc or research assistant), the institution where the mentorship took place, and the first and final mentorship years. Our analysis is based on mentorship relationships that ended between 2000 and 2013. The reason we use 2000 as the starting point of our analysis is that before that year records of mentor–mentee pairs in our data are much sparser and fluctuate significantly from year to year. We use 2013 as the final year to keep track of publications for a period of 5 years after the final mentorship year, plus an additional 5 years to allow for the accumulation of citations received by such publications.

We merged the aforementioned genealogy data with the authors’ publication records, citations and institutional affiliations by linking AFT with the Microsoft Academic Graph (MAG), one of the largest multi-disciplinary bibliographic databases. One advantage of using the MAG database is that all entities in it (i.e. scientists, institutions and publications) have already been disambiguated and associated with unique identifiers, allowing for a sequential matching between AFT and MAG authors and affiliations. The integrated AFT and MAG data have been obtained from [24], and citation information of publications authored by AFT authors is retrieved from the MAG database. Owing to our focus on mentors with some scientific impact, we have excluded from our analysis the mentorship records of mentors who received no citations during the period preceding their final mentorship years, which pertains to a marginal 3% of the total records. Mentees are included regardless of their citation counts, as long as their paired mentors in the mentorship records have received at least one citation during the corresponding period. Notably, we find that the frequency with which mentees leave academia (reported in electronic supplementary material, figure S8) to be significantly higher when their mentors lack impact, i.e. have received no citations during the years covered by our analysis (electronic supplementary material, figure S9).

### 4.2. Measures of mentor–mentee impact persistence

#### 4.2.1. Spearman rank correlation

As one of the most common measures of intergenerational persistence, it quantifies the extent to which a mentee’s impact rank tends to be associated with their mentor’s impact rank, without requiring the relationship between the two to be loglinear. This measure provides a concise summary of positional persistence, which is independent of any changes in the distribution of citations between the two generations. Therefore, it can be easily used to make comparisons across disciplines for temporal analyses.

#### 4.2.2. Pearson correlation

An alternative measure of intergenerational persistence is the Pearson correlation, which captures the correlation between the logarithmic citation impact of mentors and mentees,


(4.1) $$r=\frac{Cov(M_{t-4,t},P_{t,t+4})}{\sqrt{Var(M_{t-4,t})Var(P_{t,t+4})}},$$

where $M_{t-4,t}$ and $P_{t,t+4}$ are, respectively, the logarithmic citation impact of mentors and mentees received within a 5-year time window before and after the final mentorship year t .

#### 4.2.3. Estimating intergenerational persistence with a random walk model

The empirical transition matrices shown in figure 1*c* capture academic intergenerational persistence by estimating the probabilities of moving from one impact ranking quintile to another across mentor–mentee generations. We here use a random walk model [10] to encapsulate a mentee’s likelihood to occupy the same quintile as their mentor, or to an adjacent one, as observed in the aforementioned empirical transition matrices. Precisely, we hypothesize that the probability of finding a mentee in quintile j when their mentor was in quintile i of their impact ranking can be mathematically represented as follows:


(4.2) $$P_{ij}=e^{-\Delta_{ij}^{2}/D}/\sum_{\ell }e^{-\Delta_{i\ell }^{2}/D},$$

where $\Delta_{ij}$ is the distance between the two quintiles, with $\Delta_{ij}=|i-j|,\forall i,j$ . The parameter D governs the extent to which mentees can ‘diffuse’ through higher or lower impact ranking quintiles with respect to their mentors. To study the evolution of overall persistence over time, we calibrate the optimal value of D for cohorts of mentor–mentee pairs across each final mentorship year. This calibration is achieved by minimizing the Frobenius norm of the discrepancy between the data in the empirical transition matrices and those predicted by the random walk model. A higher optimal D value signifies a more homogeneous probability distribution in a transition matrix, indicative of higher mobility and lower persistence.

## Data Availability

The datasets generated and/or analysed during the current study along with the essential codes are available from the Zenodo Repository [25]. Supplementary material is available online [26].
